# Sensitivity and reliability of cerebral oxygenation responses to postural changes measured with near-infrared spectroscopy

**DOI:** 10.1007/s00421-019-04101-0

**Published:** 2019-02-15

**Authors:** Arjen Mol, Jeffrey H. H. Woltering, Willy N. J. M. Colier, Andrea B. Maier, Carel G. M. Meskers, Richard J. A. van Wezel

**Affiliations:** 1 Department of Human Movement Sciences, @AgeAmsterdam, Amsterdam Movement Sciences, Vrije Universiteit Amsterdam, Van der Boechorstraat 9, 1081 BT Amsterdam, The Netherlands; 2 Department of Biophysics, Donders Institute for Brain, Cognition and Behaviour, Radboud University, Heijendaalseweg 135, 6525 AJ Nijmegen, The Netherlands; 3 Artinis Medical Systems BV, Elst, The Netherlands; 4 Department of Medicine and Aged Care, @AgeMelbourne, The Royal Melbourne Hospital, The University of Melbourne, City Campus, Level 6 North, 300 Grattan Street, Parkville, VIC 3050 Australia; 5 Department of Rehabilitation Medicine, Amsterdam UMC, Vrije Universiteit, Amsterdam Movement Sciences, P.O. Box 7057, 1007 MB Amsterdam, The Netherlands; 6 Biomedical Signals and Systems, Technical Medical Centre, University of Twente, Zuidhorst Building, P.O. Box 217, 7500 AE Enschede, The Netherlands

**Keywords:** Cerebrovascular circulation, Sensitivity, Reliability, Physiology, Orthostatic hypotension, Cerebral autoregulation

## Abstract

**Purpose:**

Cerebral oxygenation as measured by near-infrared spectroscopy (NIRS) might be useful to discriminate between physiological and pathological responses after standing up in individuals with orthostatic hypotension. This study addressed the physiological sensitivity of the cerebral oxygenation responses as measured by NIRS to different types and speeds of postural changes in healthy adults and assessed the reliability of these responses.

**Methods:**

Cerebral oxygenated hemoglobin (O_2_Hb), deoxygenated hemoglobin (HHb) and tissue saturation index (TSI) were measured bilaterally on the forehead of 15 healthy individuals (12 male, age range 18–27) using NIRS. Participants performed three repeats of sit to stand, and slow and rapid supine to stand movements. Responses were defined as the difference between mean, minimum and maximum O_2_Hb, HHb and TSI values after standing up and baseline. Test–retest, interobserver and intersensor reliabilities were addressed using intraclass correlation coefficients (ICCs).

**Results:**

The minimum O_2_Hb response was most sensitive to postural changes and showed significant differences (− 4.09 µmol/L, *p* < 0.001) between standing up from sitting and supine position, but not between standing up at different speeds (− 0.31 µmol/L, *p* = 0.70). The minimum O_2_Hb response was the most reliable parameter (ICC > 0.6).

**Conclusions:**

In healthy individuals, NIRS-based cerebral oxygenation parameters are sensitive to postural change and discriminate between standing up from supine and sitting position with minimum O_2_Hb response as the most sensitive and reliable parameter. The results underpin the potential value for future clinical use of NIRS in individuals with orthostatic hypotension.

**Electronic supplementary material:**

The online version of this article (10.1007/s00421-019-04101-0) contains supplementary material, which is available to authorized users.

## Introduction

Adequate cerebral oxygenation is essential for physical and cognitive functioning (Agbangla et al. 2017; Lotte et al. 2018; Vasta et al. 2018; Kovarova et al. 2018). Cerebral oxygenation depends on blood pressure and cerebral perfusion (Krakow et al. 2000), which are challenged by postural changes, such as standing up from supine or sitting position (Kim et al. 2011). Changes in blood pressure and cerebral perfusion after standing up are counteracted by the baroreflex and cerebral autoregulation (Xing et al. 2017; Purkayastha et al. 2018). However, these systems do not fully prevent cerebral oxygenation drops after standing up in most individuals (van Lieshout et al. 2001), which may be the cause of symptoms of dizziness, impaired physical function and falls in patients with impaired blood pressure control after standing up, i.e., orthostatic hypotension (Mehagnoul-Schipper et al. 2000; Thomas et al. 2009; Bachus et al. 2018).

To discriminate between physiological and pathological cerebral oxygenation responses, physiological responses to various types and speeds of postural changes must be investigated. Near-infrared spectroscopy (NIRS) is a non-invasive and non-obtrusive method to measure cerebral oxygenation and was suggested to be valid by studies reporting the correlation of NIRS signals with fMRI BOLD signals and cerebral blood flow measured by transcranial Doppler (Smielewski et al. 1995; Huppert et al. 2006). Furthermore, NIRS is potentially useful to assess cerebral autoregulation (Steiner et al. 2009; Kainerstorfer et al. 2015). Previous studies investigated cerebral oxygenation responses using NIRS in healthy adults during head-up tilt (Houtman et al. 1999; Krakow et al. 2000; Kurihara et al. 2003), compared responses to standing up or sitting up in younger and older adults (Kawaguchi et al. 2001; Gatto et al. 2007; Edlow et al. 2010; Kim et al. 2011), compared responses to standing up with and without calf muscle tensing (Kawaguchi et al. 2001; van Lieshout et al. 2001), or determined reproducibility of responses in older adults (Mehagnoul-Schipper et al. 2001). These studies reported a cerebral oxygenation drop within 30 s after standing up. However, a comprehensive assessment of the dependence of NIRS-derived cerebral oxygenation responses on the type (i.e., standing up from supine versus sitting position) and speed of postural change (i.e., slow versus rapid standing up) is missing and the reliability of these responses has not been assessed.

This aim of this study was to investigate the sensitivity of the cerebral oxygenation response as measured by NIRS to different types and speeds of postural changes in healthy adults and to assess the reliability of these responses.

## Methods

All data generated or analyzed during this study are included in the supplementary information file of the published article.

### Subjects

Fifteen healthy young (mean age 22 years, SD 2.8; 12 male) individuals were recruited via oral and written advertisement in an undergraduate university teaching setting.

Volunteers were excluded from participation when having a history of stroke, cardiovascular or cerebrovascular diseases, cardiac arrhythmias, cardiovascular-related medication use, diabetes mellitus or orthostatic hypotension. Exclusion criteria were checked prior to study participation by completing a short survey. All procedures performed in studies involving human participants were in accordance with the ethical standards of the institutional and/or national research committee and with the 1964 Helsinki Declaration and its later amendments or comparable ethical standards. The study was approved by the Ethics Committee of the Faculty of Science of the Radboud University in Nijmegen. Informed consent was obtained from all individual participants included in the study.

### Instrumentation

NIRS signals reflecting concentration changes of cerebral oxygenated hemoglobin (O_2_Hb) and deoxygenated hemoglobin (HHb) and cerebral tissue saturation index (TSI) were continuously measured bilaterally on the forehead, approximately 2.5 cm above the eyebrows, using two Portalite systems (Artinis Medical Systems B.V., Elst, The Netherlands), each consisting of three light sources and one detector. The inter-optode distance (i.e., the distance between the light sources and the light detector) of the different light sources was 30, 35 and 40 mm. The sampling frequency was set at 50 Hz. O_2_Hb and HHb were computed using the modified Lambert–Beer law using Oxysoft (version 3.0, Artinis Medical Systems B.V., Elst, The Netherlands), calculating the differential pathway factor using the formula proposed by Scholkmann and Wolf (2013). TSI, defined as oxygenated hemoglobin as a percentage of total hemoglobin, was computed using spatially resolved spectroscopy (Suzuki et al. 1999).

To identify the start of postural change, a digital goniometer was attached to the participant’s trunk to measure its angle relative to the horizontal. Time needed to stand up was defined as the time from the beginning of the first deviation from baseline to the instance where the angle was stabilized.

Beat-to-beat mean arterial pressure, interbeat interval and cardiac output were measured to assess whether cerebral oxygenation responses to postural changes correspond to systemic cardiovascular responses, as these are considered to be a cause of cerebral oxygenation drops (Levine et al. 1994). Mean arterial pressure, interbeat interval and cardiac output were measured continuously using a photoplethysmograph with a cuff placed on the left middle finger (Finapres NOVA, Finapres Medical Systems BV, Enschede, The Netherlands). Peripheral oxygen saturation (SpO_2_) was measured to assess blood oxygenation changes during standing up. An analog reference signal containing a binary coding of time was imported in every device to enable off-line synchronization of the signals.

### Protocol

**Fig. 1:**
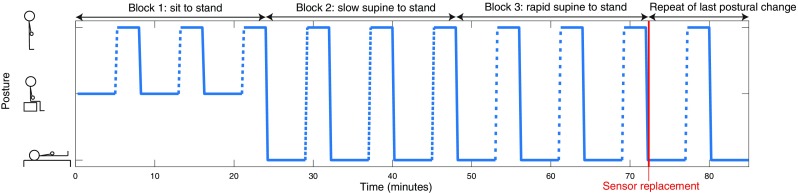
Protocol of the postural changes. The sequence of the three blocks varied among subjects due to block randomization. Each block consists of three repeats. The empty space between the dashes indicates the speed of standing up, with more space indicating higher speed

The measurements were performed in a quiet, semi-dark room with a room temperature of 21–23 °C. Three different postural changes were performed, after demonstration of the correct task execution using a short video: (1) sit to stand, defined as standing up from sitting position at a self-chosen speed; (2) slow supine to stand, defined as standing up from supine position in approximately 10 s; (3) rapid supine to stand, defined as standing up from supine position within 3 s. Subjects were stimulated to relax, instructed not to talk and asked to move as little as possible during the experiment. The three different postural changes were performed in blocks, consisting of three repetitions per block. Each repetition encompassed a 5-min resting period (supine or sitting) and a 3-min standing period (Fig. 1). The sequence of the blocks was randomized among participants to eliminate the bias due to previous postural changes. After the three blocks, the NIRS system was reapplied by a second investigator to assess the interobserver reliability. Then the last performed postural change was repeated once.

### Data analysis

NIRS, goniometer and continuous blood pressure data were synchronized and analyzed off-line using MATLAB R2017b (MathWorks, Natick, United States). NIRS and mean arterial pressure signals were filtered using a 5-s moving average filter to reduce the artifacts. Baseline values of the signals were computed as means of the 60-s period before postural change. For visualization, all signals were normalized at baseline and signals from the left and right NIRS systems were averaged. Based on previous studies reporting an early and a late oxygenation drop, the period after standing up was divided into an early and late interval, i.e., 0–30 and 30–180 s after standing up, respectively (Thomas et al. 2009; Kim et al. 2011). Parameters expressing the mean, maximum and minimum were determined for each postural change and NIRS signal for both intervals. Signal response sensitivity for postural changes was defined as the difference between these parameters and baseline.

### Statistical analysis

Statistical analyses were performed using the MATLAB R2017b statistics toolbox. Response differences between postural changes were tested using paired *t* tests. The test–retest reliability (i.e., the agreement of responses between repeats), interobserver reliability (i.e., agreement between responses before and after reapplication of the NIRS system) and intersensor reliability (i.e., agreement between responses as measured simultaneously by the left and right NIRS system) were expressed using one-way, random, single score intraclass correlation coefficients (ICCs) (McGraw and Wong 1996) and evaluated for each signal (i.e., O_2_Hb, HHb and TSI), response type (i.e., mean, maximum and minimum) and interval (i.e., 0–30 s and 30–180 s). ICC scores between 0–0.40, 0.40–0.59, 0.60–0.74 and 0.75–1 were regarded as poor, fair, good and excellent, respectively (Cicchetti 1994).

*p* values below 0.05 were considered significant. Correction for multiple comparisons was performed according to the Bonferroni method, rendering *p* values below 0.0009 significant.

## Results

The characteristics of the included individuals are listed in Table 1. Postural changes for sit to stand, slow supine to stand and rapid supine to stand were performed in 4.3 (SD 1.1), 14.4 (SD 3.9) and 6.0 (SD 1.5) s, respectively.

**Table 1:** Characteristics of the cohort

Characteristic	All (*n* = 15)
Age, years, mean (SD)	22 (2.8)
Male, *n* (%)	12 (80)
Light skin color, *n* (%)	13 (87)
Height, m, mean (SD)	1.80 (9.4)
Weight, kg, mean (SD)	71 (5.7)
Current smoking, *n* (%)	1 (6.7)
Excessive alcohol use, *n* (%)^a^	0 (0)
Resting HR, bpm, mean (SD)	75 (13)
Resting SBP, mmHg, mean (SD)	127 (7)
Resting DBP, mmHg, mean (SD)	74 (10)
Time needed for sit to stand, s, mean (SD)	4.3 (1.1)
Time needed for slow supine to stand, s, mean (SD)	14.4 (3.9)
Time needed for rapid supine to stand, s, mean (SD)	6.0 (1.5)

HR was computed as the baseline mean. Systolic blood pressure (SBP) and diastolic blood pressure (DBP) were measured using a sphygmomanometer

*SD* standard deviation, *BMI* body mass index, *HR* heart rate, *bpm* beats per minute

^a^Excessive alcohol use is defined as > 14 units per week for females and > 21 units per week for males

**Fig. 2:**
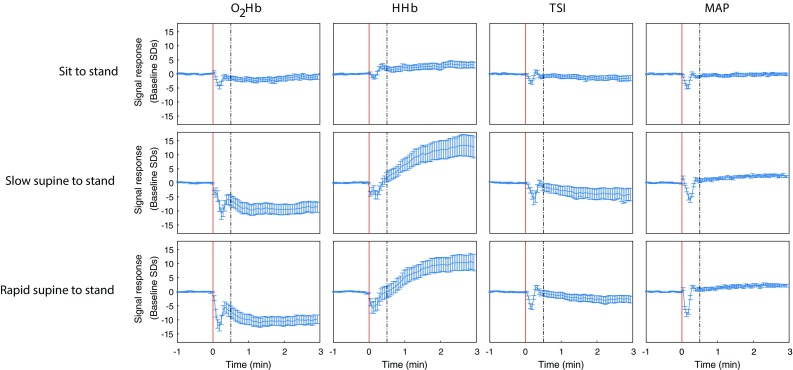
O_2_Hb, HHb, TSI and mean arterial pressure before, during and after standing up as a response to different postural changes, averaged over subjects (*n* = 15). All signals are unfiltered and normalized at baseline. The red vertical line indicates the onset of the postural change. The dashed line indicates the transition from the early (0–30 s) to the late (30–180 s) interval. The error bars indicate the standardized error of the mean

Figure 2 shows O_2_Hb, HHb, TSI and mean arterial pressure before, during and after the three types of postural change (i.e., sit to stand, slow supine to stand and rapid supine to stand), normalized at baseline and averaged over all 15 subjects. In the early interval (0–30 s), O_2_Hb, HHb and TSI showed a drop, which was most prominent in the O_2_Hb signal and in the rapid supine to stand condition. In the late interval, O_2_Hb and TSI showed a small decrease, while HHb showed a clear increase. None of the NIRS signals returned to baseline within the measurement period. Mean arterial pressure showed a pattern similar to O_2_Hb and TSI in the early interval, but remained stable in the late interval. Figure 1 in the electronic supplementary material (ESM.1) shows the cerebral oxygenation responses for the three female participants, showing similar patterns as the responses of the entire population.

Mean SpO_2_ in the early interval did not differ significantly from baseline in any type of postural change. Interbeat interval and cardiac output showed a decrease and an increase in the early interval, respectively, for any postural change, as shown in figure ESM.2.

**Fig. 3:**
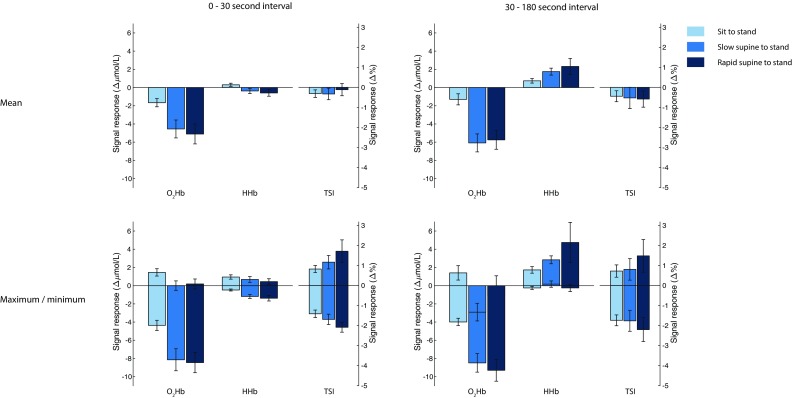
Signal response sensitivity of O_2_Hb, HHb and TSI for different types of postural changes, averaged over subjects (*n* = 15). The results are computed from the filtered signals. The upper panels depict the mean of the signal within the interval relative to baseline. The lower panels indicate the highest and lowest value (most positive and most negative bar, respectively) within the interval relative to baseline. The error bars indicate the standardized error of the mean. *O*_*2*_*Hb* oxygenated hemoglobin, *HHb* deoxygenated hemoglobin, *TSI* tissue saturation index

Figure 3 shows the O_2_Hb, HHb and TSI signal response sensitivity to three postural changes for both intervals. As shown in Table 2, the responses differed significantly between sit to stand and both slow or rapid supine to stand, but no significantly different responses between slow and rapid supine to stand were observed. After correction for multiple comparisons, only differences in O_2_Hb responses remained significant, both in the early and late interval. The largest mean arterial pressure drop after standing up was 24.0 (SD 9.8), 26.4 (SD 14.6) and 29.0 (SD 7.1) mmHg for sit to stand, slow supine to stand and rapid supine to stand, respectively, being not significantly different between conditions.

**Table 2:** NIRS response differences between postural changes

	Sit to stand versus slow supine to stand	*p* value	Sit to stand versus rapid supine to stand	*p* value	Slow supine to stand versus rapid supine to stand	*p* value
0–30 s interval						
O_2_Hb, ∆µmol/L, mean (SD)						
Mean	− 2.89 (3.60)	**0.0077**	− 3.44 (3.64)	**0.0026**	− 0.56 (2.58)	0.4169
Maximum	− 1.45 (2.44)	**0.0368**	− 1.27 (1.86)	**0.0189**	0.18 (1.54)	0.6551
Minimum	− 3.78 (3.82)	**0.0018**	− 4.09 (3.58)	0.0006*	− 0.31 (3.04)	0.6959
HHb, ∆µmol/L, mean (SD)						
Mean	− 0.67 (1.06)	**0.0279**	− 0.89 (1.26)	**0.0158**	− 0.22 (0.99)	0.4047
Maximum	− 0.27 (1.00)	0.3231	− 0.50 (0.97)	0.0642	− 0.24 (0.50)	0.0848
Minimum	− 0.71 (0.98)	**0.0143**	− 0.91 (1.24)	**0.0131**	− 0.20 (1.05)	0.4689
TSI, ∆%, mean (SD)						
Mean	0.0 (1.1)	0.9580	0.2 (1.5)	0.6058	0.2 (1.4)	0.5495
Maximum	0.3 (1.2)	0.3031	0.9 (2.4)	0.1792	0.6 (2.4)	0.3941
Minimum	− 0.3 (1.0)	0.3169	− 0.7 (1.1)	**0.0309**	− 0.4 (0.8)	0.0625
30–180 s interval						
O_2_Hb, ∆µmol/L, mean (SD)						
Mean	− 4.79 (3.78)	0.0002*	− 4.45 (3.84)	0.0005*	0.34 (3.95)	0.7451
Maximum	− 4.31 (3.71)	0.0005*	− 2.57 (8.74)	0.2739	1.74 (8.59)	0.4448
Minimum	− 4.50 (4.01)	0.0007*	− 5.31 (4.44)	0.0004*	− 0.81 (3.46)	0.3792
HHb, ∆µmol/L, mean (SD)						
Mean	1.01 (1.10)	**0.0031**	1.58 (3.06)	0.0663	0.57 (2.51)	0.3941
Maximum	1.11 (1.11)	**0.0017**	3.01 (8.13)	0.1731	1.90 (7.67)	0.3537
Minimum	0.42 (1.41)	0.2653	0.00 (1.65)	0.9956	− 0.42 (1.25)	0.2145
TSI, ∆%, mean (SD)						
Mean	− 0.1 (2.1)	0.8743	− 0.1 (1.9)	0.7806	− 0.1 (1.0)	0.8259
Maximum	0.1 (1.9)	0.8672	0.8 (3.6)	0.4273	0.7 (3.5)	0.4644
Minimum	− 0.0 (2.1)	0.9637	− 0.5 (2.4)	0.4707	− 0.4 (1.3)	0.2260

Bold values indicate significant differences before correction for multiple comparisons

NIRS responses (i.e., mean, highest value and lowest value) in two intervals, compared between postural changes. Significantly different responses were observed when comparing sit to stand with supine to stand. The responses do not differ significantly between slow and rapid supine to stand

*This association remains significant after correction for multiple comparisons

**Fig. 4:**
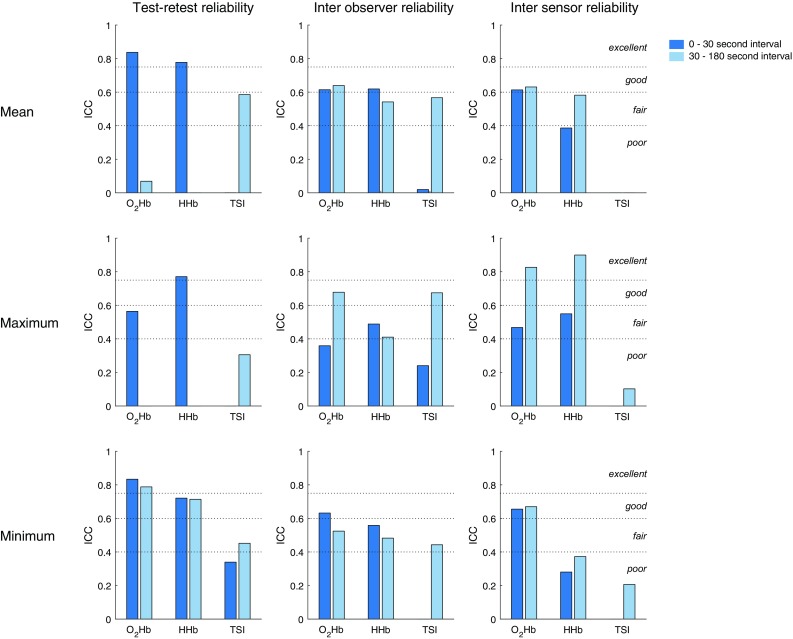
Test–retest reliability, interobserver reliability and intersensor reliability, presented as intraclass correlation (ICC), separate for each signal, response type and interval. The ICCs are computed from the filtered signals. The dotted lines delineate ICC scores regarded as excellent, good, fair and poor, as indicated in the right panels. An absent bar signifies an ICC of zero or lower. *O*_*2*_*Hb* oxygenated hemoglobin, *HHb* deoxygenated hemoglobin, *TSI* tissue saturation index

Figure 4 shows the test–retest reliability, interobserver reliability and intersensor reliability for each signal, parameter and interval. Overall, the minimum O_2_Hb response in the early interval resulted in the highest reliability scores, being good to excellent. None of the parameters derived from HHb and TSI had good or excellent test–retest, interobserver and intersensor reliability.

## Discussion

Cerebral oxygenation as measured by NIRS was sensitive to postural changes in healthy adults. Oxygenated hemoglobin (O_2_Hb) showed the most prominent drop after standing up, which was significantly different between standing up from supine and from sitting position, but not between slow and rapid standing up. Compared to other parameters, the minimum O_2_Hb response in the early interval showed good to excellent reliability, identifying this as the preferred parameter in the assessment of cerebral oxygenation responses to postural changes.

Both oxygenated and deoxygenated hemoglobin dropped in the early phase after standing up, indicating a lower concentration of total hemoglobin, therewith reflecting a decrease of cerebral perfusion. This is in line with the early perfusion drop after standing up reported by transcranial Doppler studies (van Lieshout et al. 2001; Thomas et al. 2009). This perfusion drop indicates that cerebral autoregulation may not immediately compensate for blood pressure drops resulting from gravitational pooling after standing up, even in healthy adults (Zhang et al. 2002; Chisholm and Anpalahan 2017; Xing et al. 2017; van Wijnen et al. 2017). The perfusion drop in the context of a constant brain oxygen demand is likely to be the cause of the cerebral hemoglobin saturation decrease, as reflected by the drop in TSI. Altered lung function during standing up may have contributed to the early drop in O_2_Hb and TSI after standing up. However, SpO_2_ did not show a significant drop after standing up, indicating this contribution was probably not large, if at all present. Furthermore, the decrease of interbeat interval and increase of cardiac output suggest a sufficient cardiac response to postural change, implying cardiac function does not account for the cerebral oxygenation drop.

The late, gradual drop of O_2_Hb and TSI to below baseline and rise of HHb to above baseline are consistent with previous studies (Krakow et al. 2000; Mehagnoul-Schipper et al. 2001; Kim et al. 2011) and are not likely to arise from gravitational pooling, as healthy adults usually recover blood pressure within 30 s after standing up (van Wijnen et al. 2017). These may be explained by a persistently decreased brain perfusion after standing up due to persistent hydrostatic pressure differences, as reported by transcranial Doppler studies (van Lieshout et al. 2001; Kim et al. 2011). The lower brain perfusion and a constant brain oxygen demand might cause a larger part of the available hemoglobin to become deoxygenated, thereby explaining a drop of O_2_Hb and TSI and a rise of HHb.

The significantly different O_2_Hb responses between standing up from sitting and supine position measured in the present study could not be explained by corresponding differences in blood pressure drop. Instead, these O_2_Hb response differences might be explained by dependence of cerebral autoregulation on the type of postural change, independent of the magnitude of the blood pressure drop. The vestibular system may be involved, as standing up from supine and sitting position causes different vestibular stimuli, influencing cerebral autoregulation (Serrador et al. 2009) and therewith cerebral oxygenation.

No significant differences were found between responses to rapid and slow supine to standing in the O_2_Hb, HHb and TSI signals, as would be expected from studies showing that cerebral autoregulation acts as a high-pass filter, implying that rapid blood pressure drops cannot be compensated for as adequately as slow blood pressure drops (Rickards and Tzeng 2014; Tarumi and Zhang 2018). Cerebral autoregulation may not have been tested to its maximum, as measured differences of blood pressure drops between the slow and rapid supine to stand conditions in the present population were small and not significant. Further studies in patients with impaired blood pressure control, e.g., patients with orthostatic hypotension are required.

The lower overall test–retest reliability and intersensor reliability of TSI responses compared to O_2_Hb and HHb responses may be explained by an insufficient validity of the assumptions needed to compute TSI, such as homogeneity of brain tissue (Yoshitani et al. 2007; Murkin and Arango 2009). The substantial TSI response differences, as measured by the left and right NIRS devices, suggest different tissue properties underlying both devices, e.g., differences in skull thickness, which were reported to be considerable in a recent study (Sawosz et al. 2016). Alternatively, a relatively low sensitivity of TSI to postural changes may imply that TSI parameters are relatively sensitive to noise, leading to lower TSI reliability scores.

The NIRS measurements investigated in the present study are potentially influenced by changes in scalp perfusion after standing up, which is not directly regulated by cerebral autoregulation. Studies on the contribution of scalp perfusion to cerebral oxygenation as measured by NIRS are contradictory. Cerebral oxygenation was reported to correlate significantly with jugular vein oxygenation, but not with facial vein oxygenation, suggesting signals derived from NIRS measurements primarily reflect cerebral processes (Murkin and Arango 2009). However, significant changes in TSI, as measured by NIRS, were reported after inducing scalp ischemia using a tourniquet, indicating a significant influence of scalp blood flow (Germon et al. 1994).

The subjects needed more time to stand up than instructed in both the slow and rapid supine to stand conditions. This may be attributable to underestimation of the speed of standing up by the subjects. Alternatively, it may be due to the definition of the time needed to stand up, which requires a stabilized goniometer signal. If subjects stood up sufficiently rapidly, but needed some extra time to fully stabilize, this may have prolonged the measured time needed to stand up.

### Strength and limitations

The strength of this study is that it addresses the sensitivity of cerebral oxygenation signals for different types and speeds of postural change and systematically assesses the test–retest, interobserver and intersensor reliability for various parameters. The small number of included individuals is a limitation of this study, potentially introducing sampling error and limiting study power. The majority of the included individuals were young males, potentially limiting generalizability. Furthermore, as the experiment included only one session, no conclusions can be drawn regarding the day-to-day reproducibility of the parameters, which may be important to explain the variation of cerebral oxygenation responses in healthy adults.

The results elucidate the cerebral oxygenation response to different types and speeds of postural change in healthy adults. However, they do not provide an integrative view on the cardiovascular reaction to postural change, which would contribute to the understanding of the pathophysiology of orthostatic hypotension. Future studies should address this issue, simultaneously assessing blood pressure, arterial and venous vasoreactivity, calf muscle function, sympathetic and parasympathetic function as well as cerebral oxygenation.

This study does not provide results on how to predict syncope or orthostatic symptoms, as these were not recorded in this study. However, the reported results on cerebral oxygenation changes during different types and speeds of standing up in healthy adults are necessary to determine any dependence of these responses on age in future studies and to classify future NIRS measurements in patients with orthostatic hypotension as physiological or pathological.

## Conclusion and future direction

This study demonstrates that cerebral oxygenation responses measured using NIRS are sensitive to postural change and discriminate between standing up from supine and from sitting position, but not between slow and rapid standing up in healthy adults. Furthermore, it identifies minimum O_2_Hb response in the early interval as a sensitive and reliable parameter, suggesting this parameter to be of potential value for future clinical use in older adults with impaired blood pressure control, e.g., orthostatic hypotension. Future research should address other cardiovascular responses to postural change such as arterial and venous vasoreactivity in an integrative approach. Furthermore, it should address the effect of aging on the cerebral oxygenation response to different types and speeds of postural change, and investigate the potential of NIRS to predict clinical outcomes such as falls in patients with orthostatic hypotension. In contrast to healthy adults, the speed of standing up might be important for the cerebral oxygenation response in this group of patients due to inadequate blood pressure regulation and cerebral autoregulation, warranting further research.

## Electronic supplementary material

Below is the link to the electronic supplementary material.

Supplementary material 1 (DOCX 11 KB)

Supplementary material 2 (PDF 205 KB)

Supplementary material 3 (PDF 161 KB)

## Abbreviations

HHb: Deoxygenated hemoglobin
ICC: Intraclass correlation coefficient
NIRS: Near-infrared spectroscopy
O_2_Hb: Oxygenated hemoglobin
SD: Standard deviation
TSI: Tissue saturation index
