# Supplementary material for: Sensitivity and reliability of cerebral oxygenation responses to postural changes measured with near-infrared spectroscopy
Source: Eur J Appl Physiol. 2019 Feb 15;119(5):1117–25. doi: 10.1007/s00421-019-04101-0 (PMC6469633; doi:10.1007/s00421-019-04101-0)
Supplement: Supplementary file 1 — Supplementary material 1 (DOCX 11 KB) [file 421_2019_4101_MOESM1_ESM.docx]

**ELECTRONIC SUPPLEMENTARY MATERIAL FIGURE LEGENDS**

**Fig ESM.1** **O_2_Hb, HHb, TSI and mean arterial pressure of the included female individuals before, during and after standing up as a response to different postural changes, averaged over subjects (n=3).** All signals are unfiltered and normalized at baseline. The red vertical line indicates the onset of the postural change. The dashed line indicates the transition from the early (0 – 30 seconds) to the late (30 – 180 seconds) interval. The error bars indicate the standardized error of the mean.

**Fig ESM.2** **Inter beat interval and cardiac output before, during and after standing up as a response to different postural changes, averaged over subjects (n=15).** All signals are unfiltered and normalized at baseline. The red vertical line indicates the onset of the postural change. The dashed line indicates the transition from the early (0 – 30 seconds) to the late (30 – 180 seconds) interval. The error bars indicate the standardized error of the mean.
