# Supplementary figures and images for: Sensitivity and reliability of cerebral oxygenation responses to postural changes measured with near-infrared spectroscopy
Source: Eur J Appl Physiol. 2019 Feb 15;119(5):1117–25. doi: 10.1007/s00421-019-04101-0 (PMC6469633; doi:10.1007/s00421-019-04101-0)

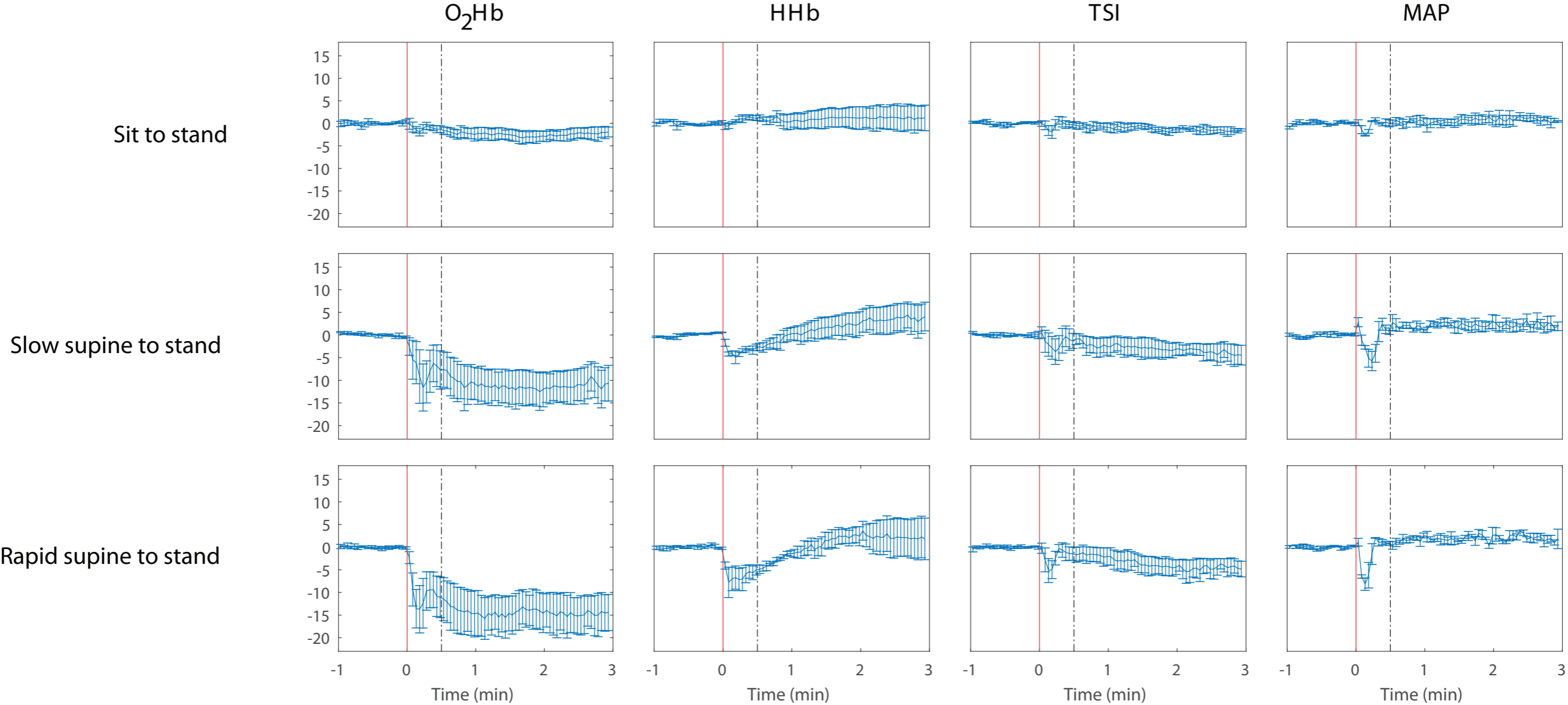

Supplement: Supplementary file 2 — Supplementary material 2 (PDF 205 KB) [file 421_2019_4101_MOESM2_ESM.pdf]

## Inter beat interval

## Cardiac output

Sit to stand

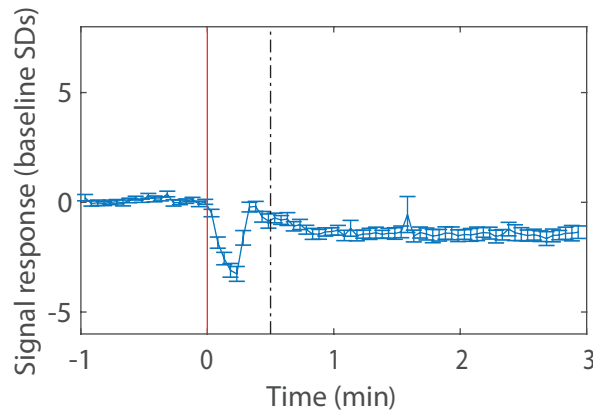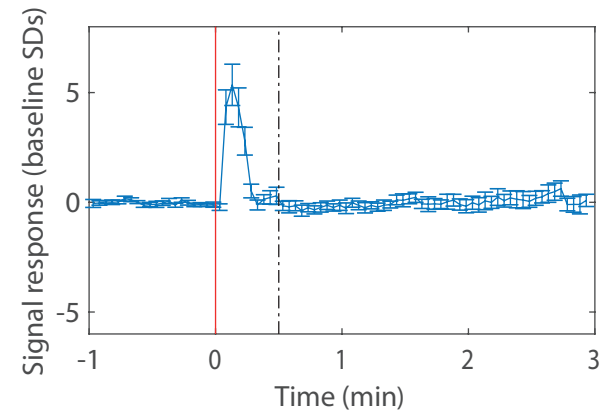

Slow supine to stand

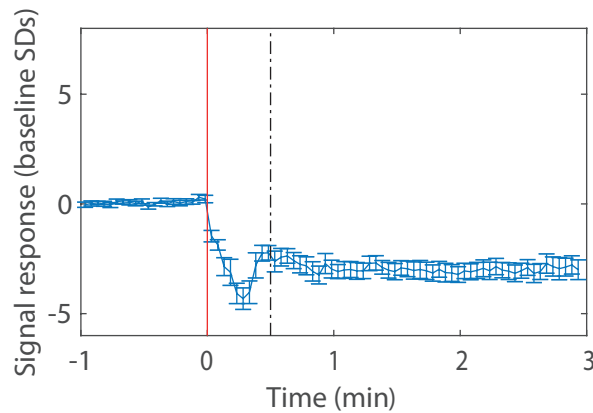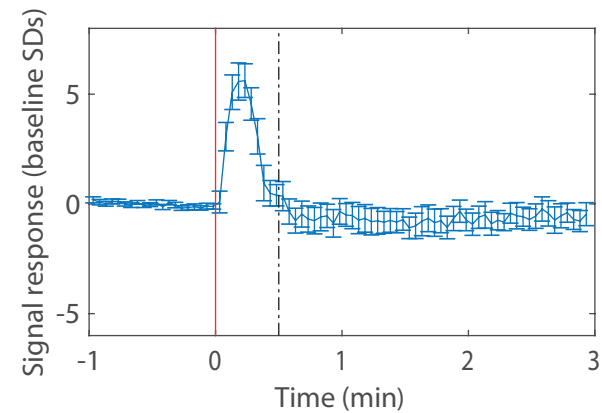

Rapid supine to stand

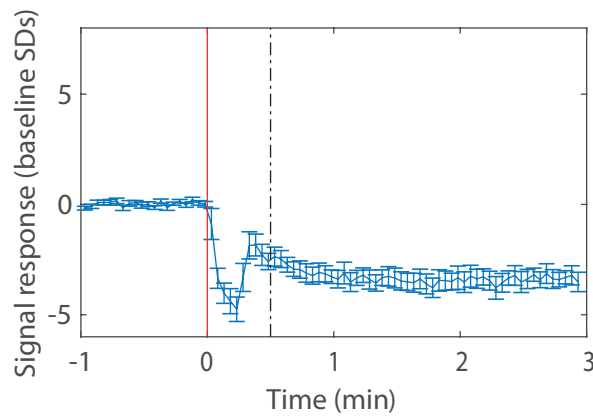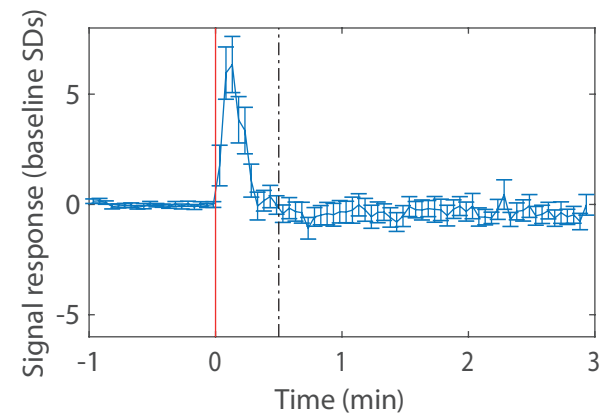

Supplement: Supplementary file 3 — Supplementary material 3 (PDF 161 KB) [file 421_2019_4101_MOESM3_ESM.pdf]
